# Robotic-Assisted Esophagectomy Leads to Significant Reduction in Postoperative Acute Pain: A Retrospective Clinical Trial

**DOI:** 10.1245/s10434-022-12200-0

**Published:** 2022-07-19

**Authors:** Jens P. Hoelzen, Karl J. Sander, Matteo Sesia, Dhruvajyoti Roy, Emile Rijcken, Alexander Schnabel, Benjamin Struecker, Mazen A. Juratli, Andreas Pascher

**Affiliations:** 1 Department of General, Visceral and Transplant Surgery, University Hospital Muenster, Muenster, Germany; 2 Department of Data Sciences and Operations, Marshall School of Business, University of Southern California, Los Angeles, CA USA; 3 Helio Health, Irvine, CA USA; 4 Department of Anesthesiology, Intensive Care and Pain Medicine, University Hospital Muenster, Muenster, Germany

## Abstract

**Background:**

Robot-assisted minimally invasive esophagectomy (RAMIE) shows promising results regarding postoperative complications in patients with esophageal cancer. To date, no data are available regarding postoperative analgesic consumption. The aim of this work is to evaluate analgesic consumption after esophagectomy.

**Methods:**

A total of 274 Ivor Lewis esophageal resections performed sequentially from January 2012 to December 2020 were evaluated. RAMIE cases (*n* = 51) were compared with the hybrid technique (laparoscopic abdominal phase followed by open thoracotomy, *n* = 59) and open abdominothoracic esophagectomy (OTE) (*n* = 164). Data were collected retrospectively. The primary endpoint was the overall postoperative morphine consumption, which represents a reliable indirect measurement of pain. Pain levels recorded on the first, third, and fifth postoperative days were assessed as secondary endpoints.

**Results:**

A total of 274 patients were included. The postoperative opioid consumption rate for patients who underwent RAMIE (quartiles: 0.14, 0.23, 0.36 mg morphine milligram equivalents (MME)/kg body weight (bw)/day) was significantly lower than in the open group (0.19, 0.33, 0.58 mg MME/kg bw/day, *p* = 0.016). The overall postoperative opioid consumption for patients who underwent RAMIE was significantly lower (2.45, 3.63, 7.20 mg MME/kg bw/day; morphine milligram equivalents per kilogram body weight) compared with the open (4.85, 8.59, 14.63 MME/kg bw/day, *p* < 0.0001) and hybrid (4.13, 6.84, 11.36 MME/kg bw/day, *p* = 0.008) groups. Patients who underwent RAMIE reported lower pain scores compared with the open group on the fifth postoperative day, both at rest (*p* = 0.004) and while performing activities (*p* < 0.001).

**Conclusions:**

This study shows that patients who underwent RAMIE experienced similar postoperative pain while requiring significantly lower amounts of opioids compared with patients who underwent open and hybrid surgery. Further studies are required to verify the results.

The incidence of esophageal cancer in the Western world has increased in recent years.^1^ Despite advances in therapy, the survival rate of this disease is low, making esophageal carcinomas the sixth leading cause of global cancer mortality.^2^ The only curative treatment of locally advanced diseases is the surgical esophagus resection, mostly in combination with neoadjuvant treatment.^3^ Complete minimally invasive and hybrid techniques are increasingly superseding conventional open surgery.^4–8^

In recent years, the significance of robot-assisted and full-robotic minimally invasive esophagectomies for the treatment process of locally advanced esophageal cancer has remarkably increased. The advanced magnified 3D overview, enhanced maneuverability of surgical tools in constrained space, seven degrees of freedom of movement, and reduced-access trauma have helped overcome prior limitations of laparoscopic minimal invasive procedures.^9^

Since the onset of using robotics in esophageal surgery, several cohort studies have indicated that robotic techniques are a viable alternative to the current conventional standard of open procedures, both in terms of safety and oncological outcomes.^10–15^ The single previous randomized study (ROBOT trial) asserted lower rates of complications and postoperative pain. ^16^ The availability of data on robot-assisted esophageal surgery is limited, and the need for new randomized research is compounded by the growing desire for robotic treatment among patients.

This study examined the primary endpoint of morphine consumption as a proxy for the hypothetical level of pain that patients would have experienced in the absence of analgesics. This endpoint is more informative than pain scores in a retrospective analysis because patients receive a pain-adjusted regime of analgesics.

Reduced operation trauma and excellent postoperative pain management can help patients avoid pain, which is a critical component of contemporary surgery. Therefore, in addition to cumulative morphine dose and pain scores, this study assessed complication rates, blood loss, and postoperative hospital and intensive care stay.

## Methods

### Study Design

The study was an investigator-driven and investigator-initiated, single-center, retrospective clinical trial of 274 patients who had an Ivor Lewis esophageal resection performed at the Muenster University Hospital (Muenster, Germany). Between 1 January 2012 and 31 October 2020, patients were treated with one of three different surgical techniques for oncological esophageal resection. The procedures carried out were robotic-assisted minimal invasive esophagectomy (RAMIE) (da Vinci Surgery System, Intuitive Surgical Inc., Sunnyvale, CA), hybrid esophagectomy (HYBRID) (laparoscopic gastric mobilization and open right thoracotomy), and complete open esophagectomy (OTE). The medical records of the patient sample were evaluated for postoperative pain and opioid usage, retrospectively. Datasets were complete for all included patients.

This study was conducted according to guidelines laid out in the Declaration of Helsinki with Good Clinical Practice (GCP), and the STROCSS 2019 Guideline.^17–19^ Additional approval was obtained from the combined ethics committee of the University of Muenster (Muenster, Germany) and the Medical Association of Westphalia-Lippe (Reference Number: 2021-051-f-S). Written general consent for scientific use of medical data was obtained from all treated patients. The study was registered in the German Clinical Trial Register (Reference Number DRKS00027859).

### Patients

The study included all patients aged ≥ 18 years with a histological diagnosis of thoracic or abdominal esophagus carcinoma, which was surgically resectable at the time of diagnosis or (if performed) post-neoadjuvant therapy. The sample includes all patients treated via Ivor Lewis esophagectomy with the above-mentioned techniques, fulfilling these criteria at the Muenster University Hospital between 1 January 2012 and 31 October 2020. Patients who demonstrated either a cervical esophageal carcinoma, a carcinoma of the gastroesophageal junction, classified as Siewert III,^20^ or intraoperative termination following diagnostic laparoscopy were excluded. After introduction of the RAMIE approach, the first 16 surgery instances were also excluded owing to the operating surgeon’s primary learning curve with structured proctoring.^11^ Two surgeons, who since 2008 have specialized in the field of upper gastrointestinal (GI) surgery, performed all the procedures studied. All robotic-assisted procedures were performed by one of these two surgeons. The learning curves of the open and hybrid technologies occurred prior to 2012 and accordingly have not been accounted for.

The diagnostic workup of all cases consisted of a physical and nutritional assessment, an endoscopy including biopsy, an endoscopic ultrasound, and a computer tomography scan for cancer staging. The indication for surgery and, if required, neoadjuvant therapy was provided by a multidisciplinary cancer board at the Muenster University Hospital (Muenster, Germany). All neoadjuvant treatment was performed following the German Cancer Society (DKG) guidelines for esophageal adenocarcinoma and squamous cell carcinoma. During the study period, neoadjuvant chemotherapy or chemoradiotherapy was conducted following the FLOT (fluorouracil/leucovorin/oxaliplatin/docetaxel) or CROSS (carboplatin/paclitaxel) schemes.^21,22^

In all three groups, postoperative pain management was based on epidural anesthesia in combination with a pain-adjusted regime of opioid and non-opioid analgesics administered orally or intravenously.^23^

Patients scheduled for Ivor Lewis esophagectomy have an epidural catheter placed at the level of the seventh to ninth vertebrae. All catheters were placed before surgery in awake patients by experienced anesthesiologists. On the intensive care unit and the general wards, the quality of the epidural anesthesia is controlled daily by an experienced acute pain service specialist. Ineffective epidural catheters were removed as early as possible or were placed again if needed.^24,25^ During the observation period of this trial, no major changes in the treatment algorithms (epidural anesthesia) of the acute pain service have occurred at our center.

Evaluation and changes of pain medication were conducted by a specialized anesthesiologic team in consultation with the surgeons. Prior to patients being discharged, all opioid analgesics were discontinued. Owing to regulatory standards, pain therapy with morphine is largely restricted to in-patient hospital use. If postoperative pain persists, the patients’ hospital stay is subsequently prolonged. The subjective sensation of pain was examined multiple times each day using a numerical rating scale. For this study, the median of these values per day and patient were evaluated.

All dissected tumors were evaluated by a specialized gastrointestinal pathologist according to a standardized protocol. The resection margins were examined intraoperatively using frozen section techniques and postoperatively using histological analysis. According to the most recent UICC classification, additional tumor extension, presence, localization, and number of lymph nodes were documented.^26,27^

Intraoperatively, chest tubes and a sponge-coated nasogastric tube connected with a negative pressure of −125 mmHg were placed in all cases. All patients were monitored in the ICU after surgery for at least one night. If there were no complications, the patient was transferred to the general ward. All patients underwent a routine check on the fifth day post-surgery for anastomotic insufficiency by conducting an esophagogastroduodenoscopy. In the case of regular findings, the nasogastric tube was removed, and an esophagogram was performed. The oral diet was started with liquid followed by soft food, each for 3 days. Physiotherapy in all groups was started at the intensive care unit 1 day after surgery and includes chairing on the first postoperative day and walking on the second postoperative day.

Chest drains were removed after exclusion of a chylothorax and a drainage output of less than 200 ml per day.

The protocols for the intensive care unit stay and further postoperative management underwent no major changes since 2012. The same assessment can be made for the diet regime, mobilization through physiotherapy, respiratory training, and blood management. There was no standardized Enhanced Recovery After Surgery (ERAS) program in any group.

### Surgical Technique

The RAMIE technique is a two-stage transthoracic esophagectomy.^28^ The abdominal stage was conducted via conventional laparoscopy. The thoracic stage was executed by using a da Vinci Surgical System (Intuitive Surgical Inc, Sunnyvale, CA).

The abdominal phase of the hybrid esophagectomy was performed laparoscopically, as with the RAMIE method. An anterolateral thoracotomy on the right side was performed during the thoracic stage. A median laparotomy was followed by an anterolateral thoracotomy in the traditional open esophagectomy.

All three surgical methods are based on the exact same technique for reconstruction of the esophagus. In all three groups in the abdominal stage, a bendable endoscopic linear cutter with a length of 45 mm is used in the preparation of the gastric tube. The anastomotic technique in the thoracic stage varied only in the surgical access route (open and hybrid: anterolateral thoracotomy, RAMIE mini-thoracotomy approximately 8 cm). In all techniques, a plate is sutured into the esophageal settlement margin via a purse-string suture. The gastric tube is anastomosed using a 29 mm CDH circular stapler (Ethicon Inc., Raritan, NJ) in end-to-side technique. The lateral part of the gastric tube with the attached tumor-bearing specimen is detached using the bendable endoscopic linear cutter (45 mm). Usually, for this step, two staple loaders (with a staple depth of 1.8–3 mm) are needed.

### Endpoints

The primary endpoint was the postoperative opioid usage in morphine milligram equivalents, measured either cumulatively, or normalized by patient weight and length of stay. The postoperative pain levels, measured on days 1, 3, and 5 during movement and rest, were documented using a numerical rating scale (NRS), which were treated as secondary endpoints. Morphine consumption is justified as a primary endpoint because this is a retrospective study in which patients received a pain-adjusted regime of analgesics. Therefore, if analgesic consumption differs across groups, subsequent pain levels are not comparable. In contrast, opioid consumption is a more reliable proxy for the level of pain, which patients would have experienced in the hypothetical absence of a pain management regime. Furthermore, minimizing opioid consumption while maintaining patient comfort is a desirable clinical goal to reduce opioid-related side effects.^29^

Additional secondary endpoints were the length of postoperative hospitalization stay, duration of ICU stay, postoperative complications, occurrence of pneumonia and anastomotic leakage, scale of blood loss, overall operation time, survival rate, and resection tumor margins. Postoperative complications were assessed following the Esophagectomy Complication Consensus Group (ECCG)^30^ using the modified Clavien–Dindo classification.^31^

### Statistical Analysis

Statistical analysis was performed using the program R version 4.0.3 (R Foundation for Statistical Computing, Wirtschaftsuniversität Wien, Vienna, Austria). Categorical variables were summarized by counts and proportions. Quantitative variables were summarized by their mean and standard deviation if normally distributed, and otherwise by quartiles. Statistical significance was determined using Fisher's combination test to assess the independence of two categorical variables, pairwise Mann–Whitney tests to compare two non-normally distributed quantitative variables, a two-sample *Z*-test to compare two proportions, a Kaplan–Meier estimator, and log-rank test to perform survival analyses. All reported *p* values were two-sided. Multiplicity adjustments for pairwise comparisons across the three groups were made through a Holm correction. The significance level for the adjusted *p* values was set to *p* = 0.050.

## Results

### Patients

Between January 2012 and October 2020, 375 patients underwent surgery for resectable esophageal cancer. Of these, 274 patients were treated by the Ivor Lewis technique and eligible for inclusion. A total of 51 patients were treated via RAMIE, 59 via HYBRID, and 164 via open surgery. In five cases, metastatic spread was found intraoperatively (two hepatic, one pulmonal, one hepatic and pulmonal, and one gastric). These cases were included in further analysis. There were no statistically significant differences in demographic and clinical characteristics among the three patient population groups at the beginning of the study (Table 1). No statistically significant difference in baseline characteristics was found between the RAMIE and the HYBRID group.

**Table 1:** Baseline characteristics (*n* = 274)

	RAMIE (*n* = 51) *n* (%)	HYBRID (*n* = 59) *n* (%)	OTE (*n* = 164) *n* (%)	*p* value
*Age, years*				*p* = 0.512
< 65	32 (62.7)	34 (57.6)	93 (56.7)	
65–75	14 (27.5)	16 (27.1)	57 (34.8)	
> 75	5 (9.8)	9 (15.3)	14 (8.5)	
*Sex*				*p* = 0.686
F	7 (13.7)	10 (16.9)	32 (20)	
M	44 (86.3)	49 (83.1)	132 (80)	
Ethnicity				
White	51 (100)	59 (100)	164 (100)	
*BMI, kg/m*^2^				*p* = 0.121
< 20	1 (2.0)	5 (8.5)	19 (11.6)	
20–30	34 (66.7)	44 (74.6)	111 (67.7)	
> 30	16 (31.4)	10 (16.9)	34 (20.7)	
*ASA score*				*p* = 0.903
1	2 (3.9)	2 (3.4)	7 (4.3)	
2	27 (52.9)	38 (64.4)	96 (58.5)	
3	22 (43.1)	19 (32.2)	59 (36.0)	
4	0 (0)	0 (0)	2 (1.2)	
*Type of carcinoma*				*p* = 0.170
Adenocarcinoma	45 (88.2)	48 (81.3)	125 (76.2)	
Squamous cell carcinoma	6 (11.8)	11 (18.7)	39 (23.8)	
*Locations of tumor*				*p* = 0.890
Upper third	0 (0)	1 (1.7)	4 (2.4)	
Middle third	3 (5.9)	4 (6.8)	15 (9.1)	
Gastroesophageal junction	48 (94.1)	54 (91.5)	145 (88.4)	
*Neoadjuvant therapy*				*p* = 0.094
Chemotherapy	20 (39.2)	16 (27.1)	39 (23.8)	
Chemoradiotherapy	24 (47.1)	37 (62.7)	90 (54.9)	
None	7 (13.7)	6 (10.1)	35 (21.3)	
*Charlson Comorbidity Index*				*p* = 0.196
2	1 (2.0)	4 (6.8)	18 (11.0)	
3	10 (19.6)	13 (22.0)	29 (17.7)	
4	11 (21.6)	17 (28.8)	54 (32.9)	
5	16 (31.4)	12 (20.3)	42 (25.6)	
6	8 (15.7)	7 (11.9)	12 (7.3)	
7	2 (3.9)	5 (8.5)	7 (4.3)	
8	2 (3.9)	1 (1.7)	1 (0.6)	
9	1 (2.0)	0 (0)	1 (0.6)	
*T-status pretherapeutic*				*p* = 0.587
T1	7 (13.7)	6 (10.2)	18 (11.0)	
T2	10 (19.6)	20 (33.9)	47 (28.7)	
T3	33 (64.7)	33 (55.9)	98 (59.8)	
T4	1 (2.0)	0 (0)	1 (0.6)	
*N-status pretherapeutic*				*p* = 0.883
N0	9 (17.6)	12 (20.3)	29 (17.7)	
N+	42 (82.4)	47 (79.7)	135 (82.3)	

*BMI* body mass index, *ASA* American Society of Anesthesiologists

### Clinical Endpoints

**Fig. 1:**
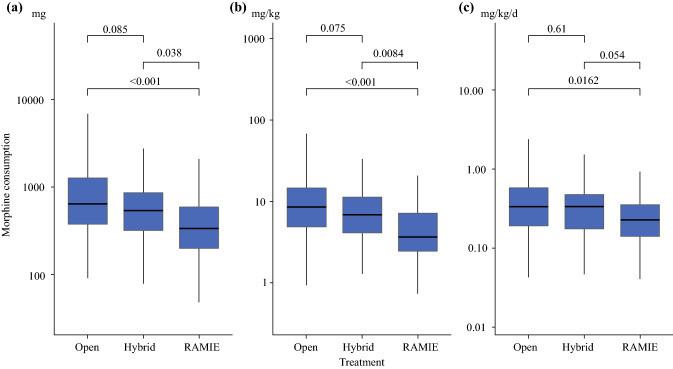
Boxplots of postoperative consumption of morphine equivalents for patients who underwent different procedures:** a** total morphine milligram equivalents, **b** morphine milligram equivalents per kilogram body weight,** c** morphine milligram equivalents per kilogram body weight per day. Pairwise *p* values comparing the distributions of morphine equivalents for different procedures were computed via Mann–Whitney tests with Holm’s correction

Patients treated by RAMIE postoperatively received significantly lower amounts of opioid analgesics (200, 335, 591 mg morphine equivalent; quartiles) compared with those treated with HYBRID (320, 540, 866 mg; *p* = 0.038) and OTE (380, 641, 1275 mg; *p* < 0.001) approaches (Figure 1). The analysis of the morphine dose adjusted by the body weight of the patients yielded a similar result [RAMIE 2.45, 3.63, 7.20 mg/kg versus HYBRID 4.13, 6.84, 11.36 mg/kg (*p* = 0.008) versus OTE 4.85, 8.59, 14.63 mg/kg (*p* < 0.001)], and, although less significantly, so did the analysis adjusted by body weight and length of stay [RAMIE 0.14, 0.23, 0.36 mg/kg/day versus HYBRID 0.18, 0.33, 0.48 mg/kg/day (*p* = 0.054) versus OTE 0.19, 0.33, 0.58 mg/kg/day (*p* = 0.016)].

**Fig. 2:**
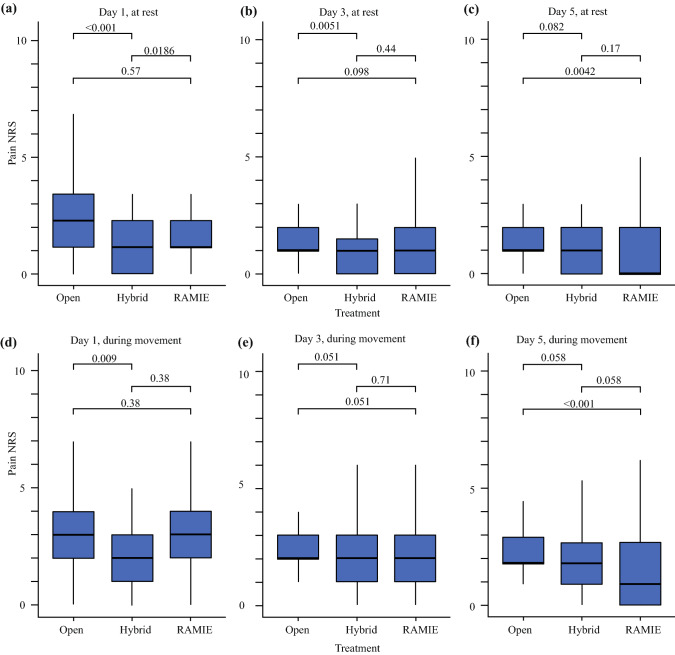
Boxplots of postoperative pain scores (NRS) reported by patients who underwent different procedures: **a**–**c** pain at rest after 1, 3, and 5 days; **d**–**f** pain during movement after 1, 3, and 5 days. Pairwise *p*-values comparing the distributions of pain scores for different procedures are computed via Mann–Whitney tests with Holm’s correction

On the first postoperative day, pain levels while active and at rest were lower in the HYBRID group compared with OTE procedures (rest: *p* <0.001; during movement: *p* = 0.009). The same results could also be evaluated at rest on the third postoperative day (*p* = 0.005). On the fifth postoperative day, pain levels while active and at rest were lower in the RAMIE group compared with OTE procedures (rest: *p* = 0.004; during movement: *p* < 0.001). All other pairwise comparisons between pain levels on the first, third, and fifth postoperative days were not statistically significant (Fig. 2).

### Postoperative Outcomes

Anastomotic leakage occurred less frequently in patients who underwent robotic (23.5%) and hybrid (22.0%) surgery than in the OTE comparison group (37.2%), although these differences were not statistically significant. The severity of complications, recorded via Clavien–Dindo classification, was lower in the RAMIE [0.0, 2.0, 3.0 modified Clavien–Dindo classification (MCDC); quartiles] and HYBRID (0.0, 2.0, 3.0 MCDC) than in the OTE (1.0, 2.5, 3.0 MCDC) group, but these differences were not statistically significant. The prevalence of severe complications (CD ≥ 3b) was lower in the RAMIE (13.7%) and HYBRID (11.9%) than in the OTE group (21.3%), but these differences were not statistically significant (Table 2).

**Table 2:** Postoperative statistics (*n* = 274)

	RAMIE (*n* = 51)	HYBRID (*n* = 59)	OTE (*n* = 164)	*p* value		
				RAMIE–OTE	RAMIE–HYBRID	HYBRID–OTE
Morphine equivalent dose total, mg*	335 (200, 591)	540 (321, 866)	641 (380, 1272)	*p* < 0.001	*p* = 0.038	p = 0.085
Morphine equivalent dose total/kg bw, mg/kg*	3.63 (2.45, 7.20)	6.84 (4.13, 11.36)	8.59 (4.85, 14.63)	*p* < 0.001	*p* = 0.008	p = 0.075
Morphine equivalent dose total/kg bw/day hospital stay, mg/kg/day*	0.23 (0.14, 0.36)	0.33 (0.18, 0.48)	0.33 (0.19, 0.58)	*p* = 0.016	*p* = 0.054	*p* = 0.614
Complications, MCDC*	2.00 (0.00, 3.00)	2.00 (0.00, 3.00)	2.50 (1.00, 3.00)	*p* = 0.142	*p* = 0.598	*p* = 0.035
Severe complication, MCDC ≥ 3b [*n* (%)]	7 (13.7)	7 (11.9)	35 (21.3)	*p* = 0.639	*p* = 0.996	*p* = 0.482
Pneumonia [*n* (%)]	11 (21.6)	15 (25.4)	47 (28.7)	*p* = 0.123	*p* = 0.292	*p* = 0.852
Anastomotic leakage^a^ [*n* (%)]	12 (23.5)	13 (22.0)	61 (37.2)	*p* = 0.206	*p* = 0.221	*p* = 0.150
Type I (conservative)	0 (0)	0 (0)	0 (0)			
Type II (nonsurgical intervention)	9 (17.6)	9 (15.3)	37 (22.6)			
Type III (surgical intervention)	3 (5.9)	4 (6.8)	24 (14.6)			
Reoperations [*n* (%)]	6 (11.8)	6 (10.2)	28 (17.1)	*p* = 0.983	*p* = 0.739	*p* = 0.875
Hospital stay, days*	18 (13, 24.5)	18 (15, 32)	22 (16, 46)	*p* = 0.002	*p* = 0.117	*p* = 0.117
ICU stay, days*	3 (2.5, 6)	7 (5, 16.5)	10 (7, 18)	*p* < 0.001	*p* < 0.001	*p* = 0.005
Epidural anesthesia, days*	5 (5, 7)	6 (5, 7)	6 (5, 7)	*p* = 0.479	*p* = 0.226	*p* = 0.392
Time until bowel movement, days*	4 (3, 5)	4 (3, 5)	4 (3, 5)	*p* = 0.494	*p* = 0.722	*p* = 0.752
30-Day mortality [*n* (%)]	0 (0)	1 (2)	3 (2)	*p* = 1.000	*p* = 1.000	*p* = 1.000

^*^Median (interquartile range)

^a^Complications graded according to Low et al.

*bw* body weight, *MCDC* modified Clavien–Dindo classification, *ICU* intensive care unit

### Intraoperative Outcomes

The operative time was significantly higher in the robotic (454, 513, 610 min; quartiles) compared with hybrid (300, 329, 364 min, *p* < 0.001) and open (274, 307, 358 min, *p* < 0.001) groups. However, the duration of intensive care stay was significantly lower among patients undergoing RAMIE (2.5, 3.0, 6.0 days) compared with the HYBRID (5.0, 7.0, 16.5 days, *p* < 0.001) and OTE (7.0, 10.0, 18.25 days, *p* < 0.001) groups. The duration of postoperative hospital stay was significantly lower in the RAMIE group (13.0, 18.0, 24.5 days) than in the OTE (16.0, 22.3, 46.1 days, *p* < 0.001) group, but similar to that of the HYBRID group (15.1, 18.2, 32.1 days).

The reported blood loss in RAMIE (0, 0, 300 ml) and hybrid operations (0, 100, 500 ml) was lower than in open procedures (200, 500, 800 ml) (both *p* < 0.001). No significant difference was found in between RAMIE and HYBRID (*p* = 0.14). Conversions occurred in neither RAMIE nor the laparoscopic part of the hybrid technique (Table 3).

**Table 3:** Intraoperative and pathological Statistics (*n* = 274)

	RAMIE (*n* = 51)	HYBRID (*n* = 59)	OTE (*n* = 164)	*p* value
Operating time, min*	513 (454.5, 610.5)	329 (300, 364)	306.5 (274, 358)	RAMIE–OTE < 0.001 RAMIE–HYBRID < 0.001 HYBRID–OTE *p* = 0.024
Blood loss, ml*	0 (0, 300)	100 (0, 500)	500 (200, 800)	RAMIE–OTE *p* < 0.001 RAMIE–HYBRID *p* = 0.144 HYBRID–OTE *p* = 0.001
Conversions [*n* (%)]	0 (0)	0 (0)	NA	
Conversion thorax	0 (0)	NA	NA	
Conversion abdomen	0 (0)	0 (0)	NA	
Pathological T-status [*n* (%)]				*p* = 0.827
T0	11 (21.6)	12 (20.3)	27 (16.5)	
T1	10 (19.6)	15 (25.4)	46 (28.0)	
T2	6 (11.8)	7 (11.9)	27 (16.5)	
T3	24 (47.1)	25 (42.4)	62 (37.8)	
Pathological N-status [*n* (%)]				*p* = 0.662
N0	28 (54.9)	35 (59.3)	100 (61.0)	
N1	8 (15.7)	11 (18.6)	33 (20.1)	
N2	10 (19.6)	6 (10.2)	19 (11.6)	
N3	5 (9.8)	7 (11.9)	12 (7.3)	
Radicality of surgery [*n* (%)]				RAMIE–OTE *p* = 0.594 RAMIE–HYBRID *p* = 0.292 HYBRID–OTE *p* = 0.292
R0	49 (96.1)	50 (84.7)	152 (92.7)	
R1	2 (3.9)	9 (15.3)	12 (7.3)	

^*^Median (interquartile range)

### Oncological Outcomes

The presence of pathologic verified residual tumor tissue was lower in the RAMIE (3.9%) than in the HYBRID (15.3%) and OTE group (7.3%). However, these differences were not statistically significant. Survival rates did not differ across the three groups.

## Discussion

Patients who underwent robotic surgery consumed significantly fewer opioid analgesics to achieve a sufficient analgesia than the other two groups (RAMIE versus HYBRID *p* = 0.038; RAMIE versus OTE *p* < 0.001). We found a significant difference regarding the per day amount of morphine consumption, when comparing RAMIE with open surgery techniques. Between RAMIE and HYBRID techniques, a significant difference can only be evaluated in the total morphine consumption and not for per day consumption, as stated. However, there is no significant difference between the duration of hospital stay for these groups, which allows comparability of the total amount of morphine consumption. Distortions of the reported morphine equivalent doses due to different lengths of epidural anesthesia were ruled out as no significant difference was observed.^32^

Effective postoperative pain control is relevant for the patient without causing severe opioid-related adverse events, because a fast decrease of postoperative pain intensity is associated with a reduced risk of postoperative chronic pain.^33,34^ Overall, pain intensities at rest and especially with activity, which is much more relevant as a patient-specific postoperative pain outcome parameter,^35^ remained low in all groups. This highlights the efficacy of epidural analgesia in patients undergoing open, hybrid, or robot-assisted esophagectomy. More specifically, patients in the HYBRID group reported less pain on the first postoperative day than those in the OTE group, both at rest (*p* < 0.001) and while active (*p* = 0.009). In patients who underwent robotic-assisted operation, lower pain scores were found in the analysis on the fifth postoperative day compared with the OTE group both at rest (*p* = 0.004) and during movement (*p* < 0.001). However, these differences in postoperative pain intensity were only small and are possibly not clinically relevant. Nevertheless, this study clearly highlights that patients undergoing invasive and long-lasting laparoscopic robot-assisted abdominal procedures (like esophagectomy) still might be better treated with epidural catheters instead of single shot regional analgesia [such as transversus abdominis plain (TAP) blocks] or even no regional analgesia combined with opioids and non-opioids. However, our data are retrospective, and this finding should be therefore interpreted with caution. This is in contrast to a recently published statement regarding the future of epidural analgesia in laparoscopic abdominal surgery.^36^ From our point of view, it is premature to abandon general epidural analgesia in patients undergoing laparoscopic abdominal procedures; instead, the effectiveness and safety of epidural analgesia (compared with systemic analgesia or single-shot peripheral regional analgesia) should be studied specifically in patients undergoing long-lasting, invasive laparoscopic abdominal procedures in the future.

Apart from postoperative pain intensity, the observed difference in opioid consumption between open, hybrid, or robot-assisted esophagectomy was large and is clinically relevant owing to the decreased risk for opioid-related adverse events. The lower postoperative opioid analgesic consumption in patients who underwent RAMIE may be partly explained by a lower operative trauma associated with the robotic surgical technique. Owing to the retrospective study design, a detailed evaluation of adverse effects caused by opioid therapy was not possible with our data. However, low opioid consumption must be considered as a surrogate parameter for lower nociceptive activation. Further prospective studies are needed to address these findings.

Both RAMIE and HYBRID patient groups lost significantly less blood during surgery than those operated on openly (RAMIE versus OTE *p* < 0.001; HYBRID versus OTE *p* = 0.001). Even though considerably lower blood loss was measured with RAMIE, there was no significant advantage in the comparison with the HYBRID group.

Another possible factor contributing to the lower analgesic consumption of RAMIE is the lower severity of complications and anastomotic leakages compared with those operated on openly, although these differences are not statistically significant. Compared with data from other centers, anastomotic insufficiency rates were overall on the higher end. ^37–39^ After surgery, all patients received a postoperative esophagogastroduodenoscopy in which the anastomosis and the gastric tube were evaluated. This preventive intervention potentially resulted in a higher detection rate of anastomotic leakage, as even clinically not manifested defects were reported according to Esophagectomy Complications Consensus Group standards.^30^

Severe postoperative complications (MCDC ≥ 3b) occurred less often in RAMIE (13.7%) and HYBRID (11.9%) than in OTE (21.3%) groups, although no statistically significant difference was proven. Similar findings were established for differences in reoperation rate (RAMIE 11.8% versus HYBRID 10.2% versus OTE 17.1%; *p* > 0.05).

Patient recovery depended highly on mobilization and respiratory training. Possible complications, especially respiratory complications like pneumonia, can occur when mobilization is restricted owing to pain.^40,41^ Even though no statistically significant influence is evaluated, a trend toward lower occurrence of pneumonia in minimal invasive procedures can be seen in the data (RAMIE 21.6% versus HYBRID 25.4% versus OTE 28.7%) as described in the wider literature.^42,43^ This retrospective evaluation established typical clinical symptoms and the start of antibiotic therapy (MCDC 2) as the critical diagnostic criterion for pneumonia since confirmation by microbiological sputum examination was frequently omitted in clinically clear cases. This resulted in an overall higher rate of this complication.

On analysis, there was a significant increase in operating time with robotic surgery (RAMIE 454, 513, 610 min) compared with both the HYBRID (300, 329, 364; *p* < 0.001) and OTE groups (274, 307, 358; *p* < 0.001). Although a decrease in operation time can be expected in the future owing to the long learning curve of about 70 patients in RAMIE,^44^ data from established centers show an advantage of open surgery here.^16^

The length of postoperative hospital stay for patients undergoing RAMIE (13.0, 18.0, 24.5 days) was significantly lower compared with the OTE group (16.0, 22.3, 46.1 days, *p* < 0.001), and similar to the HYBRID group (15.1, 18.2, 32.1 days). This fits in with the findings of lower postoperative analgesic consumption, as both interdependent factors suggest a faster reconvalescence.^45^

Since there is no strict separation of the intensive care and observation ward, patients stay longer during routine observation and in the case of complications. Therefore, the length of stay recorded at this unit is overall high.^46^ Nevertheless, a significant reduction for patients undergoing robotic-assisted operation compared with both other surgical groups was observed (OTE *p* < 0.001; HYBRID *p* < 0.001).

In a comparison of the HYBRID with the OTE group, patients undergoing hybrid techniques showed lower values for the postoperative length of stay as well as for the intensive care unit stay, although only the second difference was statistically significant (hospital *p* = 0.059; ICU *p* = 0.005).

The external validity of these results is limited because the length of hospital and intensive care unit stay are prolonged at our center by confounders as economic, structural, and health policy factors. However, the relative reduction between the groups can be interpreted as a sign of a faster reconvalescence.

When comparing the RAMIE, hybrid, and open techniques, the biggest differences are regarding the complete minimally invasive approach. This advantage is lacking in the other two techniques but is not exclusive to robotic-assisted procedures. We cannot conclusively say whether our results are due to robotic or minimal invasive advantages as one is dependent on the other. Therefore, we are awaiting the results of the ROBOT-2 trial (ClinicalTrials.gov ID: NCT04306458), which started in January 2021 and compares full robotic to full laparoscopic approach in a randomized multicenter design study.^47^

We acknowledge certain limitations to this study. The single-center design might influence the generalizability and transferability of the results. Even so, the collected data and analyses are consistent to other published findings. Owing to the nonrandomized study design, it cannot be ruled out that patients' pain perception may be affected by the placebo effect leading to a reduced analgesic consumption. Additionally, a similar bias can be assumed for the treatment team as expectations on pain intensity may vary depending on the surgical approach. The data used in this study are routine data collected several times a day and not study-specific data. Therefore, it cannot be assumed that the patient was influenced either intentionally or unintentionally.

Since the procedures examined in this study were performed by only two different surgeons, there are few confounders that could have altered the results. However, owing to limits in documentation, these differences in technical nuances of the operation and postoperative patient management within the framework of the hospital standards may have affected the outcome. As with most retrospective studies, time period bias cannot be excluded in this clinical trial. The results should be verified by reproduction as a randomized controlled approach, although a randomization is inherently difficult, as a non-neglectable number of patients already requested robotic intervention in the ROBOT trial.^16^ This number is expected to increase with further scientific proof of the feasibility of the approach. The strength of this evaluation is based on the large-scale sample size of 274 patients, all of them with complete datasets.

## Conclusions

Patients undergoing esophagectomy benefit from robotic surgery as compared with hybrid or open techniques owing to a significant lower need for analgesics to treat postoperative pain. In addition, patients who underwent RAMIE had significantly less intraoperative blood loss. Despite longer operation times, patients who underwent RAMIE experienced a shorter stay in the intensive care unit and accelerated postoperative recovery. Further studies, preferably randomized trials, should be conducted to further verify the shown benefits of the RAMIE approach.
